# Foreword to the Special Issue

**DOI:** 10.21542/gcsp.2017.17

**Published:** 2017-10-31

**Authors:** Jane Burns

**Affiliations:** Professor and Director, Kawasaki Disease Research Center, Dept. of Pediatrics, University of California, San Diego, 9500 Gilman Dr., La Jolla, CA 92093-0641, USA

This year marks the 50th anniversary of the description of the publication in Japanese of Dr. Kawasaki’s landmark series of 50 children suffering from the condition that would later bear his name (Kawasaki, 1967). The fact that Kawasaki felt the need to gather 50 subjects before publication attests to the uphill battle that he waged in getting the medical establishment in Japan to recognize that he was describing a new clinical entity (Burns et al., 2000). Kawasaki had no knowledge of the potentially dire consequences of the condition that he described, as it was not until 1970, when the Japanese Ministry of Health conducted a questionnaire survey of Japan, that the 10 deaths from acute myocardial infarction were discovered. By the time of his seminal publication in English in 1974, the complications of coronary artery vasculitis and aneurysm formation were clearly linked to the clinical illness (Kawasaki et al., 1974).

Even before the 1974 publication in English, it was recognized that Kawasaki disease (KD) existed outside of Japan. In the early 1970s, Marian Melish and Raquel Hicks, two assistant professors at the University of Hawaii, kept meeting at the bedside of patients on whom they were asked to consult. These children were suffering from a mysterious condition involving fever and mucocutaneous features that defied classification in known disease categories. Melish learned from a visiting Japanese physician of cases of an emerging disease in Japanese children that shared features with their new disease among Asian American children in Hawaii. After corresponding with Kawasaki - Melish, Hicks, and the pathologist Eunice Larson reported their cases of KD in 1974 at the Society for Pediatric Research annual meeting (Melish et al., 1974).

In this special issue of *Global Cardiology Science and Practice*, we celebrate the 50th anniversary of the original case series and honor Kawasaki’s vision and courage. This issue includes, for the first time, case series of children suffering from KD in Russia and Egypt. Recognition of the “globalization” of KD has been a gradual process, which was likely aided by the waning of rheumatic fever and measles in many developing countries. KD may already have surpassed rheumatic fever as the most important cause of acquired pediatric heart disease in India and Egypt. Unfortunately, in many of the countries with growing numbers of cases, the standard treatment with intravenous immunoglobulin is simply not available or not affordable. Let us hope that in the near future a better understanding of KD and its pathogenesis will lead to improved, more affordable treatments and ultimately, to prevention of this potentially devastating disease.

## References

[ref-1] Burns JC, Kushner HI, Bastian JF, Shike H, Shimizu C, Matsubara T, Turner CL (2000). Kawasaki disease: A brief history. Pediatrics.

[ref-2] Kawasaki T (1967). [Acute febrile mucocutaneous syndrome with lymphoid involvement with specific desquamation of the fingers and toes in children]. Arerugi.

[ref-3] Kawasaki T, Kosaki F, Okawa S, Shigematsu I, Yanagawa H (1974). A new infantile acute febrile mucocutaneous lymph node syndrome (MLNS) prevailing in Japan. Pediatrics.

[ref-4] Melish ME, Hicks RM, E L (1974). Mucocutaneous lymph node syndrome in the US. Pediatric Research.

